# Investigation of the impact of supplemental reflective films to improve micro-light climate within tomato plant canopy in solar greenhouses

**DOI:** 10.3389/fpls.2022.966596

**Published:** 2022-08-23

**Authors:** Anhua Liu, Michael Henke, Yiming Li, Yue Zhang, Demin Xu, Xingan Liu, Tianlai Li

**Affiliations:** ^1^College of Horticulture, Shenyang Agricultural University, Shenyang, China; ^2^Key Laboratory of Protected Horticulture, Ministry of Education, Shenyang, China; ^3^National & Local Joint Engineering Research Center of Northern Horticultural Facilities Design & Application Technology (Liaoning), Shenyang,China; ^4^Plant Sciences Core Facility, CEITEC-Central European Institute of Technology, Masaryk University, Brno, Czechia; ^5^Leibniz Institute of Plant Genetics and Crop Plant Research (IPK), Stadt Seeland, Germany; ^6^College of Engineering, Shenyang Agricultural University, Shenyang, China

**Keywords:** in-silico light simulation, passive light supplement, micro-light climate, reflective film, GroIMP

## Abstract

The non-uniform growth and development of crops within Chinese Solar Greenhouses (CSG) is directly related to the micro-light climate within canopy. In practice, reflective films are used to improve micro-light climate within plant canopy by homogenizing light distribution and so increasing total plant light interception. However, as to our knowledge, the contributions to light distribution within canopy have not been investigated for passive reflector like reflective films. Field experiments dealing with light conditions and growth behavior over time, are complicated to carry out, time-consuming and hard to control, while however, accurate measurements of how reflective films influence the micro-light climate of canopy are an essential step to improve the growth conditions for any crop. Here, we propose a supplementary light strategy using reflective films to improve light distribution within plant canopy. Based on the example of CSG, a 3D greenhouse model including a detailed 3D tomato canopy structure was constructed to simulate the influence of supplementary reflective films to improve micro-light climate. Comparison of measured solar radiation intensity with predicted model data demonstrated that the model could precisely predict light radiation intensity over time with different time points and positions in the greenhouse. A series of reflective film configurations were investigated based on features analysis of light distribution in the tomato canopy on sunny days using the proposed model. The reflective film configuration scheme with the highest impact significantly improved the evenness of horizontal and vertical light distribution in tomato canopy. The strategy provided here can be used to configure reflective films that will enhance light conditions in CSG, which can be applied and extended in different scenarios.

## Introduction

Chinese solar greenhouses (CSG) are constructed to provide adequate growth condition for vegetable production even during hard winter times without any auxiliary heating or additional light sources. CSG represent an important guarantee for China’s annual vegetable supply balance (Tong et al., 2013) and significantly contribute to increase farmers’ income (Panwar et al., 2011; Guo et al., 2012). According to the Agricultural Mechanization Management Department of China (2018), by the end of 2018, CSG cover 577 thousand hectares. High-efficiency utilization of solar radiation is the most essential ingredient in the extensive use of CSG. Sunlight enters the CSG via the transparent south roof and influences various microenvironmental factors inside the CSG, including canopy illumination, temperature, and humidity (Papadopoulos and Hao, 1997). Therefore, solar radiation intensity and distribution in crop canopy are of paramount importance for crop production and the major factor one can influence to improve light conditions within CSG. However, due to the interaction of CSG’s unique structure and plant architecture, the light distribution within canopy is usually uneven, resulting in non-uniform crop growth. This problem has restricted sunlight use efficiency, and impeded the standardization process of crop management and production.

Many studies have been conducted regarding light interception optimization of solar greenhouses from different perspectives. Some of them have reported the optimization of architectural parameters of solar greenhouses to improve overall light interception inside, including cross-sectional parameters of the greenhouse (Chen et al., 2019; Esmaeli and Roshandel, 2020), shape and angle of the south roof (Cabrera et al., 2009; Tong et al., 2013), and orientation of the greenhouse (Chen et al., 2018). Even when the overall light interception inside the greenhouse increases, the shading problem caused by enclosures and the plants themselves remains unresolved in CSG. Other studies investigated the use of auxiliary facilities including artificial lighting and reflective materials to improve light environment (Glenn and Puterka, 2007; Kumar et al., 2016; Muneer et al., 2019; Katzin et al., 2021). Artificial lighting systems such as light emitting diodes (LEDs), usually present a low surface temperature and produce less radiant heat (Zhang et al., 2017). This technology has shown its suitability to improve the illumination of top, interior or lateral surface of tall plants (Gomez et al., 2013), providing a good solution to non-uniform light distribution inside greenhouses. Moreover, benefits of LEDs were manifested both in crop quality and yield (Monostori et al., 2018; Weaver et al., 2019). For example, Tewolde et al. (2016) found that the use of daytime LED inter-lighting significantly increased tomato yield by 27% and nighttime LED inter-lighting increased 20% the total soluble solids and 25% ascorbic acid content of tomato fruits in winter. However, at the same time, those facilities with supplemental lighting consume more than twice the amount of energy and produce three times the amount of carbon dioxide compared to greenhouses without supplemental lighting (Raaphorst et al., 2019). With LED technologies rapid evolvement in energy use efficiency (Hemming et al., 2019), LED light sources currently have 60% higher efficiency than traditional assimilation lighting at converting electrical power to photo-synthetically active radiation and this value is promising to rise (Katzin et al., 2021). In other words, LEDs are expected for further reducing energy consumption, investments and costs in the future.

Another powerful method to improve micro-light climate is installing reflective materials with low price inside greenhouse (Kong and Meng, 2004). Reflective materials can improve crop canopy light interception by reflecting sunlight that otherwise would be intercepted in the non-planting areas as ground or side walls of a greenhouse back to the crop canopy. Especially the light interception improvement of plants close to the reflectors is obvious (Gupta and Tiwari, 2005; Sethi and Arora, 2009; Tang et al., 2015). Numerous field studies have demonstrated benefits of reflective materials generated by increasing crop light interception such as decreasing the incidence of tomato yellow leaf curl disease (Sirigu et al., 2011), increasing amounts of bioactive components in blueberries (Muneer et al., 2019), strawberry yield (Rhainds et al., 2001) and apple fruit quality (Overbeck et al., 2013). These studies on reflective films are carried out from the perspective of improving total solar radiation in the growing area. To the best of our knowledge, there are no studies on the improvement of crop canopy light distribution by reflective films. Further, only a few reports have investigated the effects of the configurations of reflective materials on light intensity in the greenhouse (Cui et al., 2005; Kong et al., 2008). Kong et al. (2008) derived equations based on the principles of light reflection to optimize the height and angle parameters of the reflective board hanging below the roof ridge in an inclined solar greenhouse. Cui et al. (2005) adjusted the hanging height of the reflective film on the rear wall according to the relationship between the height angle of the sun at noon and the elevation angle of the rear roof. However, the detailed light distribution within canopy under different reflective film configurations remains unknown.

A number of studies have been carried out, investigating the light and micro-light environment within greenhouses (Cossu et al., 2018; Zhang et al., 2020a,b). The methodologies that were first used to analyze greenhouse microclimate greatly depended on real tests, which are typically a very time- and labor-consuming process. Unfortunately, these tests are only able to provide limited data. One possible method to facilitate the study of light environments are in-silico simulation using specialized simulation software. De Visser et al. (2014) optimized illumination and light quality for glasshouse for tomato production in the Netherlands with additional LED light supply. Zhang et al. (2021) estimated canopy leaf physiology of tomato plants grown in a solar greenhouse based on simulations of light and thermal microclimate. These investigations have introduced physics-based ray-tracing techniques and considered mechanical interactions between the greenhouse and the 3D structure of plants, which could predict microclimate at the organ-level.

The purpose of this study was to configure reflective films from the perspective of improving light distribution within canopy in CSG. For this purpose, we took a major vegetable crop (tomato) as model plant, whose growth and development is closely linked to horizontal and vertical light distribution in canopy. Then, we built a coupled solar radiation-solar greenhouse-tomato population model considering the optical features and morphological data of the greenhouse and tomato plants. The model accuracy was evaluated in the presence and absence of reflective films. Further, light distribution features of tomato canopy on sunny weather in the greenhouse were determined using this model. Based on that, the effects of different reflective film configuration parameters, i.e., usage time, position and height, on light distribution in tomato canopy could be investigated and an optimal configuration could be identified. Finally, the improvement effects of the proposed configuration on light distribution of tomato canopy were investigated. The presented study offers theoretical supports for the reasonable use of reflective films for light distribution improvement within canopy in CSG. Our supplementary light strategy provides new insights for decision-making purposes about appropriate reflective film configurations in CSG.

## Materials and methods

### Modeling

In the presented study, interactive plant modelling environment GroIMP (Hemmerling et al., 2008; Kniemeyer, 2008) was used to perform all simulations. The constructed model consists of three separate models, the CSG model, the tomato plant population model, and the solar radiation model.

#### Reference greenhouse setting

**Figure 1:**
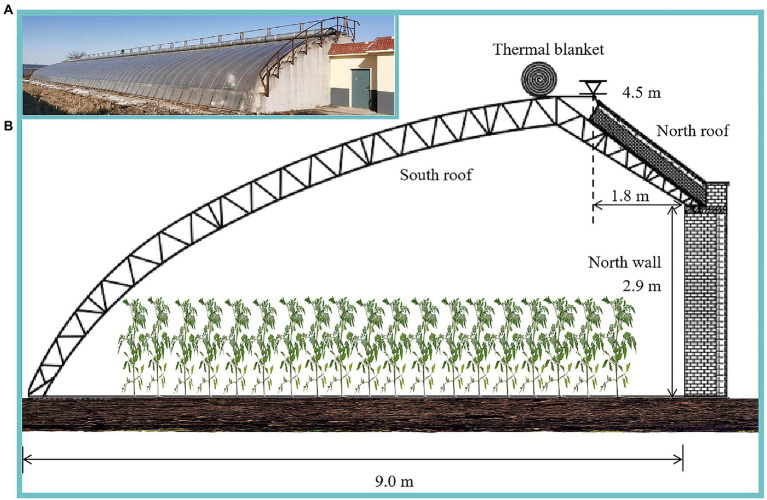
**(A)** Photograph of a real CSG taken at Shenyang Agricultural University (Photograph by Anhua Liu), and **(B)** schematic diagram of the greenhouse cross section with structural parameters.

We considered a typical standard CSG covering an area of 540 m^2^, with a width of 9 m and a length of 60 m. The north wall height was 2.9 m, and the ridge height of the greenhouse was 4.5 m. The horizontal projection of the north roof was 1.8 m. The south roof was covered with a 0.15 mm polyolefin (PO) thin film (Figure 1). The curve of the greenhouse lighting surface was fitted using Equation (1):


(1) $$Y=16.8644X^{0.5009}+0.2$$

where *X* represents the horizontal coordinate of the lighting surface; *Y* is the vertical coordinate of the lighting surface, both in cm. The greenhouse is located in Shenyang Agricultural University (41.8°N, 123.6°E), oriented south and 7° to west.

#### Growth conditions and reconstruction of tomato plants

A conventional, large-fruited tomato variety, “Liaoyuanduoli,” was adopted as the test material. Seeds were placed in petri dishes between sterile filter papers soaked in tap water for 3 days at 28°C. After germination, germinated seeds were placed in 50-well seedling plates filled with 2:1:1 (v/v/v) mixture of garden soil, humus and sand and placed at the case greenhouse. When plantlets grew 3–4 leaves, these plantlets were transferred in loam soil of the case greenhouse. The conventional cultivation mode for CSG with double rows on a broad ridge was used. The planting spacing, width of narrow and wide row, and ridge height were 0.35, 0.4, 1, and 0.1 m, respectively. The final planting density was 2.815 plants/m^2^. The plants were pruned timely, with a single branch after pruning. Tomato plants were watered 2 times every week, at 3- and 4-day intervals, with tap water through drip irrigation. Before transplanting, soil was rototilled and 110 t/ha of decomposed organic manure (cow and chicken manure) were broadcasted uniformly as basal fertilizer in the soil. After transplanting, 300.0 kg N, 150 kg P_2_O_5_/ha and 150 kg K_2_O/ha of fertilizers were applied 1 or 2 times every month with irrigation events. The case greenhouse, with windows closed, were unventilated to prevent heat loss during cold winter, with air temperature ranged from 10 to 34°C. No auxiliary heating and lighting were provided in the whole production process.

**Figure 2:**
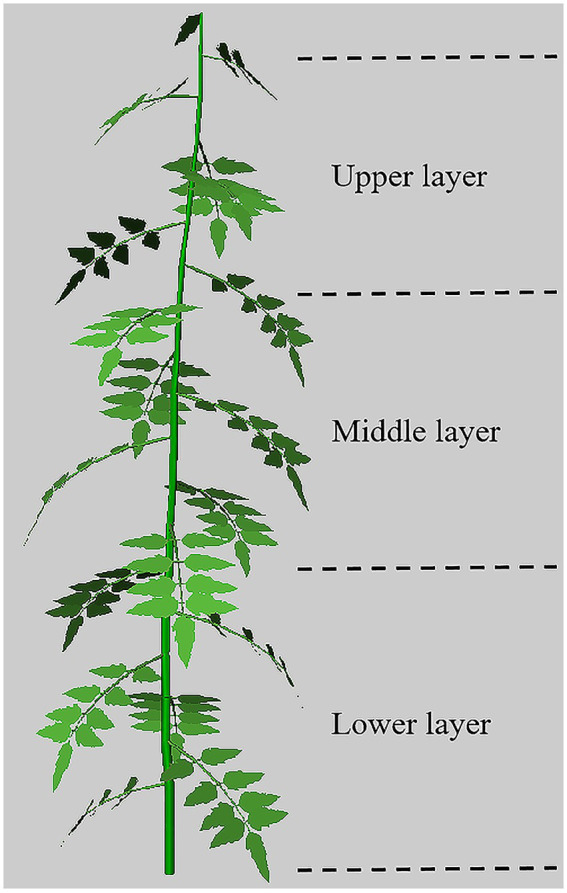
Snapshot of a single tomato plant with 18 internodes and leaves, having a total length of 1.8 m, divided into three layers: upper, middle, and lower layer, each of a layer of 6 leaves.

The first step to create a virtual tomato plant was to digitalize the structure of real tomato plants which were in the fruit setting period. Three mature tomato plants, i.e., the 5th, 10th and 15th plant from southern side to northern side, were picked out in the 10th and 30th row from eastern side to western side of the greenhouse, respectively. Different morphological parameters, including plant length, number of leaves, and petiole inclination angle, were measured manually on six mature tomato plants using a ruler and a protractor under no reflective films in the case greenhouse. A photograph of a functional leaflet was imported into ImageJ software (US National Institutes of Health, Bethesda, Maryland, United States) to determine the coordinates of a set of representative landmarks along the leaflet outline and inner shape. In the second step, the obtained coordinates are triangulated and used as input for the polygon mesh that is used as leaflet model. All virtual leaflets were assumed having the same area. Additional data on size, shape and connection sequence of other tomato organs, i.e., internodes and petioles, were also entered into plant model. A screenshot of the final virtual tomato morphology is displayed in Figure 2. 760 virtual tomato plants were integrated into the virtual greenhouse model. The general parameters of the 3D virtual greenhouse model and the tomato plant population model are summarized in Table 1. Optical properties of different components in the case greenhouse are summarized in Supplementary Table S1.

**Table 1:** General parameters of the virtual greenhouse and the virtual tomato plant population model.

Description	Value range	Unit
Greenhouse dimensions		
Lighting roof (L, W, H)	30, 7.2, 0.00015	meter
Wall (L, W, H)	30, 0.48, 2.9	meter
Ground (L, W, H)	30, 9, 0.5	meter
Roof (L, W, H)	30, 2.435, 0.2	meter
Plant arrangement		
Width of the wide plant row	1	meter
Width of the narrow plant row	0.4	meter
Tomato plant spacing	0.35	meter
Number of rows [South–North]	40	–
Number of plants per row	19	–
Tomato plant		
Maximal leaf rank per plant	18	–
Averaged plant length	1.8	meter
LAI of a single tomato plant	3.3	–
Averaged horizontal angle of petiole	55	°
Averaged vertical angle of petiole	10	°
Range of internode diameter linear interpolated from bottom to top	[0.0043, 0.0115]	meter

#### Construction of the solar radiation model

**Figure 3:**
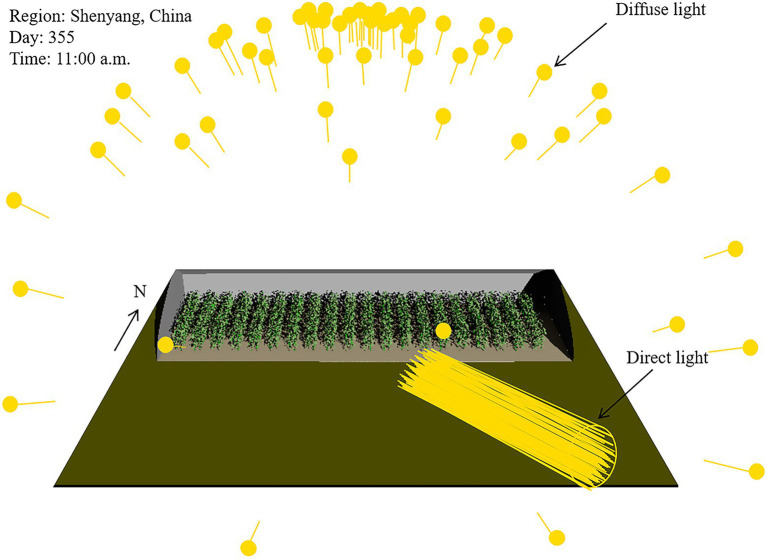
Snapshot of the virtual greenhouse model with a tomato plant population of 760 plants arranged in 19 horizontal rows and 40 vertical columns. Indicated in yellow, the position of the light sources of the diffuse light sky and the direct sun module generating together the solar radiation scenario for the scene.

The greenhouse light environment was simulated following the method reported by Buck-Sorlin et al. (2011). Briefly, the greenhouse model is surrounded by a virtual sun and sky model. The sun travels along a specific path across the sky, providing direct incident light at different geographical locations, i.e., latitudes, and times, i.e., hour of day. The diffuse sky is represented as a hemisphere of 72 directional light sources, pointing towards the center of the hemisphere, delivering diffuse light scenario. The integrated ray tracer in GroIMP was used to simulate the light scenarios (Hemmerling et al., 2013). For the simulations, 200 million virtual rays and 10 reflections per ray were used to deliver stable and reproducible results (Henke and Buck-Sorlin, 2017). A snapshot of the 3D scene, including the solar radiation, greenhouse, and tomato plant population, is shown in Figure 3.

### Analysis of light interception, light interception improving ratio and uniformity of light distribution in tomato canopy

A typical tomato leaf is composed of irregularly-shaped, pinnately compound leaves. The output results of GroIMP corresponded to light interception values for each compound leaf. The light interception values of all compound leaves of the entire tomato plant were added up to obtain the total light interception for a tomato plant. Subsequently, the total light interception data of every tomato plant in the greenhouse were entered into Origin software (OriginLab Corporation, Northhampton, MA, United States) to obtain the horizontal distribution nephogram of the tomato canopy. Similarly, the canopy of a 1.8 m long tomato plant was divided into lower, middle, and upper layers with each layer of 6 leaves (Figure 2). The light interception values of all compound leaves in each of the three canopy layers were integrated to calculate the vertical light distribution nephograms of different tomato canopy.

Light interception improving ratio in this study represents the ratio of light absorption per leaflet under some reflective film configuration to the situation without the configuration. For example, light interception improving ratio under 1.4 m high reflective film on the west side at 9:00 a.m. equals the ratio of light absorption per leaflet under 1.4 m high reflective film at 9:00 a.m. to light absorption per leaflet without 1.4 m high reflective film on the west side at 9:00 a.m.

Coefficients of variation, i.e., the ratio of standard deviation to average of a set of data, was used to evaluate the uniformity of light distribution in tomato canopy. For example, coefficients of variation under 1.4 m high reflective film equals to the ratio of standard deviation of a set of light absorption per leaflet in according tomato canopy under 1.4 m high reflective film to the average of the set of data.

### Model evaluation

**Figure 4:**
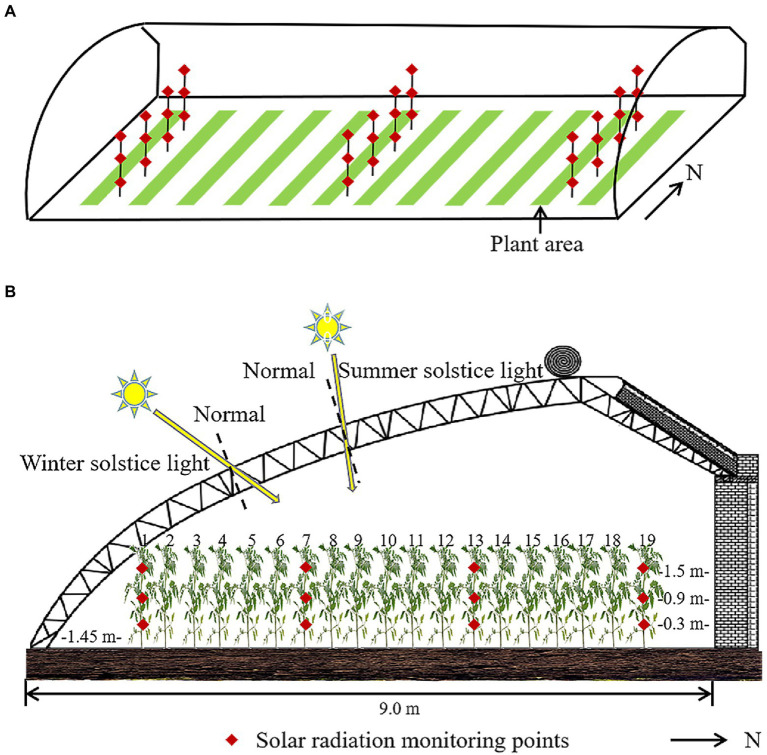
Layout of the measuring points of **(A)** the whole greenhouse, and **(B)** specific every measuring row. Same monitoring points were used for the scenes with and without reflective films. Solar radiation measuring points are at 0.3/0.9/1.5 m high close to the 1st, 7th, 13th, 19th tomato plant from south to north.

We evaluated the accuracy of our model by comparing simulated against measured data. The measurement was done on a sunny day, i.e., December 3rd, 2021, day 337 of the year, every hour between 9 a.m. and 16:00 p.m. A MP-200 pyranometer (Apogee Instruments, Inc., Logan, UT, United States) was used to measure the solar radiation intensity at different monitoring points without reflective films. This pyranometer reports the results within a temporal resolution of 1 ms. The spectral range of the pyranometer is 280–1,120 nm, with a measuring range of 0 ~ 1999 W m^−2^ and a calibration error of ±5%. One row of plants was chosen on the east, west and middle positions of the greenhouse, and the specific monitoring points of each row were shown in Figure 4. Measurements were performed at heights of 0.3, 0.9, and 1.5 m, with 12 horizontal measuring points per height. The solar radiation intensities at different positions per height of the tomato canopy were averaged. The root mean square error (RMSE) and R^2^ between the simulated and measured values were calculated.

**Figure 5:**
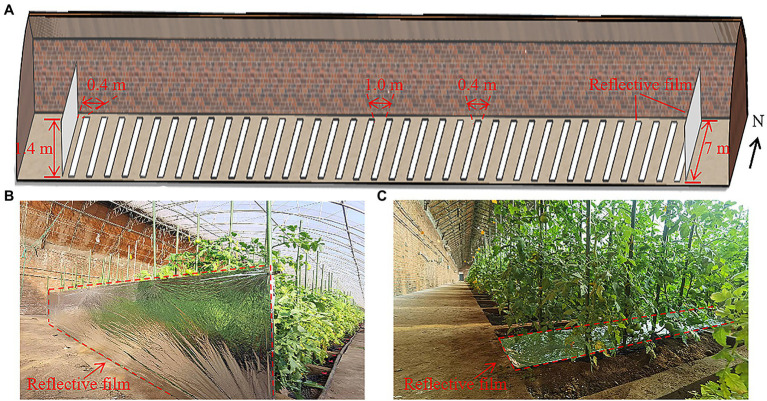
**(A)** Schematic diagram of the location and size of reflective films in model evaluation, and photographs of real scenarios with reflective films placed **(B)** on the west side of the greenhouse, and **(C)** beneath the canopy.

In order to directly compare the solar radiation intensity in the tomato canopy with and without reflective films, we measured light intensity in the same greenhouse very quickly, with firstly installing reflective film and then removing the film. Moreover, the same monitoring points, measurement ways and data analysis were used for these two scenarios (Figure 4). The positions of the reflective films are shown as in Figure 5. Reflective films with a height of 1.4 m were installed on the east and west sides of the greenhouse at a distance of 0.4 m from the plant. Reflective films with a width of 0.4 m (equal to the width of a narrow row) were installed on the ground surface between two adjacent plant rows on the same broad ridge.

### Types of simulations

After model evaluation, we first simulated light distribution patterns of tomato canopy on sunny days in low light season. Then we simulated the effects of different reflective film configurations, i.e., different usage time and height of reflective films on the west side, east side of greenhouse and under tomato canopy, on tomato canopy light distribution. Finally, we simulated light distribution under the final reflective film configuration scheme compared to the scenario without reflective films.

## Results

### Model validation

**Figure 6:**
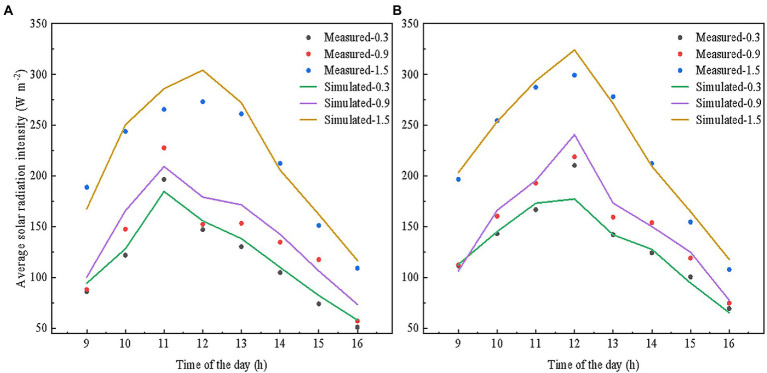
Measured versus simulated solar radiation intensities at 0.3/0.9/1.5 m heights **(A)** without, and **(B)** with reflective films on December 3rd, 2021. The measured and simulated solar radiation intensity at different positions per height were averaged, respectively, for comparison.

Figure 6 shows that the simulated and measured solar radiation intensities presented a consistent variation trend over time. As shown in Table 2, RMSE between the simulated and the measured values were between 8.11 and 17.07. This represents 2–5% of the solar radiation intensity scope of the tomato canopy. In addition, R^2^ was 0.87–0.97, which indicated the reliability of our model to simulate the dynamic distribution of solar radiation intensities at different heights of the tomato canopy with and without reflective films.

**Table 2:** Root mean square error (RMSE) and R-squared of the simulated and measured data of average solar radiation intensity at different heights (0.3/0.9/1.5 m).

		Measured/Simulated		
		0.3 m	0.9 m	1.5 m
Without reflective film	RMSE	8.11	17.07	16.61
	R-squared	0.96	0.87	0.91
With reflective film	RMSE	12.23	9.97	11.02
	R-squared	0.91	0.95	0.97

### Light distribution patterns of tomato canopy on sunny weather in low light season

**Figure 7:**
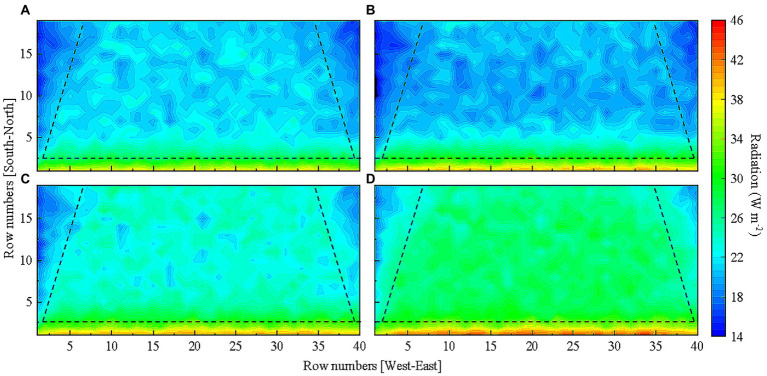
Total tomato canopy light distribution in a solar greenhouse on **(A)** November 6th, **(B)** December 30th, **(C)** January 29th, and **(D)** February 26th. Tomato canopy light interception in different areas of the greenhouse is separated by dashed lines for comparison.

Since reflectors typically work better on sunny days (Pang, 2004), light distribution patterns within tomato canopy were investigated on sunny days in low light season, i.e., November 6th, December 30th, January 29th, and February 26th in typical meteorological year (Meteorological Information Center, 2005). The ratio of daily direct solar radiation to global solar radiation ranged from 69 to 75% on these 4 days (Supplementary Table S2). Figure 7 shows the horizontal light distribution of total tomato canopy based on the light absorption per leaflet on sunny weather. Our results indicated light distribution patterns in tomato canopy were similar on sunny days in low light season. Light interception by tomato plants of the two rows located on the south side of the greenhouse was higher as compared to the rest of the plants (Figure 7). The light interception of tomato plants on the east and west sides of the greenhouse was the lowest (Figure 7).

**Figure 8:**
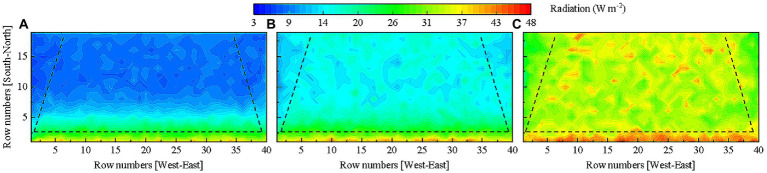
Light distribution in the **(A)** lower, **(B)** middle, and **(C)** upper layers of the tomato canopy on December 30th in the greenhouse without reflective films. Tomato canopy light interception in different areas of the greenhouse is separated by dashed lines for comparison.

As similar horizontal light distribution patterns in the tomato canopy were observed on sunny weather, December 30th with the lowest light interception was chosen as a representative day to further determine vertical light distributions in the lower, middle, and upper layers of the canopy in overwintering tomato plants. The simulation results displayed in Figure 8 shows that light interception by the two rows of tomato plants on the south side of the greenhouse was significantly higher as compared to those in other rows in every canopy layers. Light interception by plants on the east and west sides of the greenhouse was significantly lower with respect to those located in the middle of the greenhouse. The shading caused by the top canopy and inter-plant shielding significantly decreased light interception by tomato leaves present in lower positions of the plants. Light interception by leaves in the lower layer of the canopy accounted for less than 1/5 of the total.

### Effects of different reflective film configurations on tomato canopy light distribution in CSG

The effects of usage time, position, and height for reflective films on tomato canopy light distribution on December 30th were set and studied based on the light distribution features of the tomato canopy without reflective films. A 0.4 m wide reflective film was installed below the canopy between two adjacent rows on every wide ridge. Reflective films on the east and west sides of the greenhouse were installed at a distance of 0.4 m from the plants in order to save space and avoid plant contact. The reflective films on the east and west sides of the greenhouse and under the canopy were laid from the third row on the south to the north, parallel to the side walls (refer to reflective film layout shown in Figure 5).

#### Effects of usage time for reflective films on tomato canopy light interception

**Figure 9:**
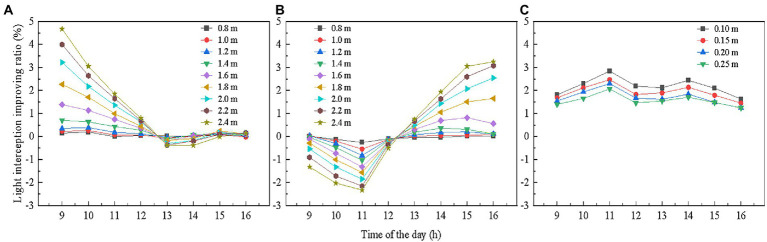
Effects of usage time of reflective films with different heights located on **(A)** the west side, **(B)** the east side of the greenhouse, and **(C)** under the canopy on tomato canopy light interception. The reflective films on the west and east sides of the greenhouse were placed on the ground, and the height of the reflective films refers to the length of the reflective films in the vertical direction. The reflective films below the canopy were placed near the plant roots, and the height of the reflective films refers to the height from the ground.

First, we explored the usage time for reflective films installed on the west side of the greenhouse. In order to eliminate the effects of reflective film height on simulation results, we examined the variations of total light interception by the tomato plant population under different reflective films heights over time. As shown in Figure 9A, when the height of the reflective film was not higher than 1.4 m, it hardly contributed to improving light interception. When the film height was above 1.4 m, it contributed to improving light interception and light interception presented a similar variation pattern over time under different reflective film heights. The light interception was all increased from 9:00 a.m. to 12:00 a.m., while the increase percentage of light interception gradually decreased over time. From 13:00 p.m. to 16:00 p.m., the use of reflective films decreased light interception by the tomato plants. Also, the decrease percentage diminished over time until reaching around zero. Hence, the usage time of reflective films on the west side of the greenhouse was 9:00 a.m. to 12:00 a.m.

We then investigated the usage time of reflective films installed on the east side of the greenhouse. As shown in Figure 9B, the effects of the reflective films on light interception of the tomato plants varied over time in an opposite manner as compared to those on the west side of the greenhouse. From 9:00 a.m. to 12:00 a.m., the use of reflective films reduced light interception of tomato canopy, while between 13:00 p.m. to 16:00 p.m., the light interception improved to varying degrees.

Figure 9C presents the relationship between the usage time of reflective films installed at different heights under the canopy and the total tomato canopy light interception. According to the results, from 9:00 a.m. to 16:00 p.m., the total light interception all increased with reflective films installed at different heights. It was interesting to note that the curve corresponding to variation of total light interception by the tomato canopy with respect to time presented two peaks. At 12:00 a.m., the increase percentage of light interception was slightly lower. This probably occurred because the solar elevation angle at midnoon presented the highest value. As the sunlight falls on the plant canopy, the shading caused by plants restricted reflective effects of the reflective films.

#### Effects of the height of reflective films on tomato canopy light interception

**Figure 10:**
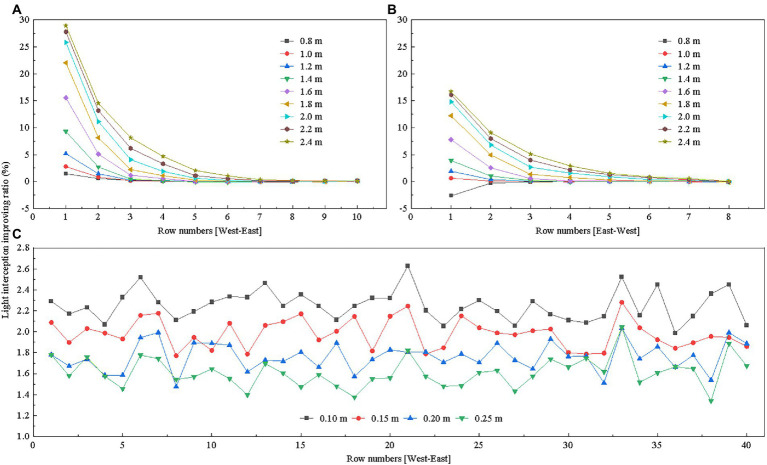
Effects of reflective film height on tomato canopy light interception: **(A)** west side, **(B)** east side of the greenhouse, and **(C)** under the canopy. The reflective films on the west and east sides of the greenhouse were placed on the ground, and the height of the reflective films refers to the length of the reflective films in the vertical direction; the reflective films below the canopy were placed near the plant roots, and the height of the reflective films refers to the height from the ground.

We examined the effects of different heights of reflective films installed on the west side of the greenhouse on tomato canopy light interception. As Figure 10A shows, improving ratio of tomato canopy light interception was positively correlated with the height of the reflective films. The reflective films should not be higher than 2.4 m due to the shape limitation of the south roof. When the reflective films were at 2.4 m high, the increased percentage of light interception by each row of plants varied between 29 and 0% from west to east. The light interception improved for five consecutive plant rows. The smaller the distance to the reflective film, the greater the effect of supplemental light.

Figure 10B shows the effects of reflective films of different heights installed on the east side of the greenhouse on light interception by a few rows of neighboring plants. Results indicated that as the height of the reflective films increased, light interception also increased. When the reflective film was at 2.4 m high, light interception improved for five plant rows from east to west. The increased percentage of light interception varied between 17 and 0% in each row.

We also examined the effects of different heights of reflective films under canopy, in combination with both 2.4 m high reflective on the west side and east side, on tomato light interception in each row. Results in Figure 10C showed that reflective films installed at different heights improved light interception with close improving ratio. The lower the distance between reflective films and the ground surface, the greater the percentage increase of light interception by each row. When the reflective film was installed at 0.10 m above ground, the reflective film was installed at the level of the ridge surface, the largest light interception percentage increase in a single row was about 2.6%.

#### Effects of the height of reflective films on tomato canopy light distribution

**Figure 11:**
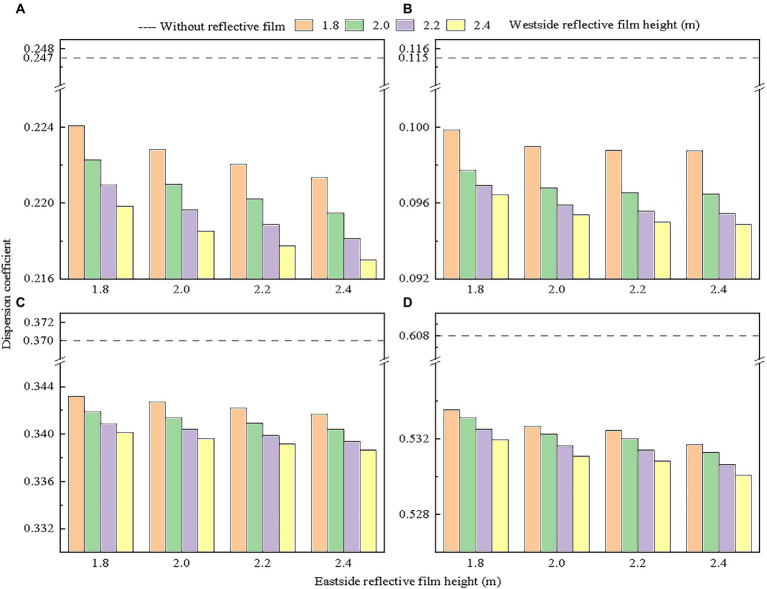
Effects of height of reflective films located on the east and west sides of the greenhouse on light distribution of: **(A)** total canopy, **(B)** upper canopy, **(C)** middle canopy, and **(D)** lower canopy compared to scenario without reflective films. The height of the reflective film under canopy was fixed at 0.1 m.

After quantifying the relationship between reflective film height and canopy light interception, we introduced the coefficient of variation to assess the effects of reflective film height on light distribution in the tomato canopy. The above studies have confirmed that light interception values were lower in the lower, middle, and upper layers of the canopy on the east and west sides of the greenhouse on sunny weather in low light season (Figure 8). The overall light interception of the lower layer of the canopy accounted for less than 20% of the total canopy light interception (Figure 8). Considering that laying reflective films under the canopy increased total light interception by less than 3% (Figure 10C), a significant difference was still observed between light interception by the lower layer of the canopy and that by the middle and upper layers of the canopy. The height of the reflective film installed under the canopy was set as 0.1 m. Four height levels of 1.8, 2.0, 2.2 and 2.4 m (no lower than plant height) were set for reflective films on the east and west sides of the greenhouse. Coefficients of variation for these 16 combinations were examined to reduce unnecessary data processing. The results in Figure 11 shows that the uniformity of overall light distribution and the light distribution in each layer of the canopy were significantly improved by using reflective films. The greater the height of the reflective films on the east and west sides of the greenhouse, the smaller the coefficient of variation and the greater the evenness of light distribution in the canopy. The best uniformity in canopy light distribution was observed when 2.4 m high reflective films were placed on the east and west sides of the greenhouse and reflective films were installed at 0.1 m height from ground surface under the canopy. The final reflective film configuration scheme is presented in Table 3.

**Table 3:** Final reflective film configuration scheme.

Position	Usage time	Height (m)
West side	9:00–12:00 a.m.	2.4
East side	13:00–16:00 p.m.	2.4
Under canopy	9:00–16:00	0.1

### Evaluation of compound gain of the final reflective film configuration scheme

**Figure 12:**
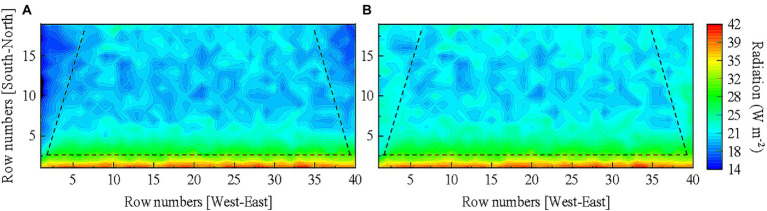
Comparison of the horizontal light distribution of tomato canopy **(A)** without reflective film and **(B)** under the final reflective film configuration.

**Figure 13:**
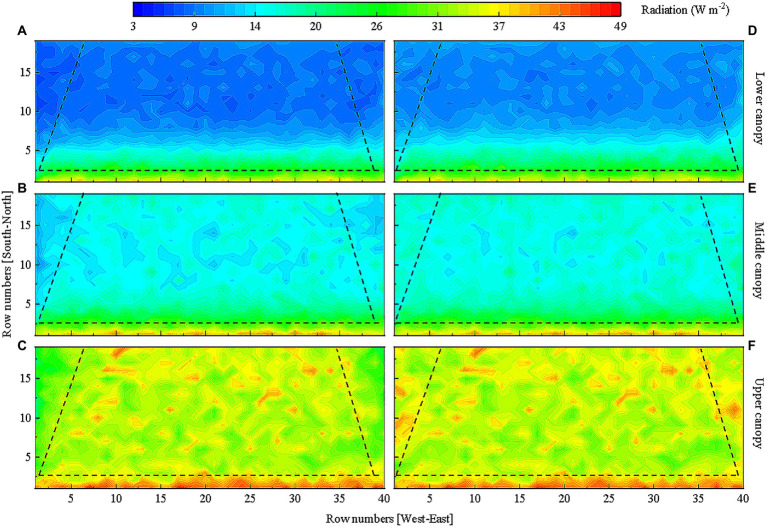
Comparison of the vertical light distribution. The simulated light distribution in the **(A)** lower, **(B)** middle, and **(C)** upper layers of tomato canopy without reflective films. The simulated light distribution in the **(D)** lower, **(E)** middle, and **(F)** upper layers of tomato canopy under final reflective film configuration scheme. Tomato canopy light interception in different areas of the greenhouse is separated by dashed lines for comparison.

Compound gain of light interception was achieved in the tomato canopy as reflective films were installed on the west and east sides of the greenhouse as well as on the ground surface. Figure 12 shows the horizontal light distribution of tomato canopy under the final reflective film configuration as well the scenario with no reflective films. Comparison of Figures 12A,B shows that the horizontal tomato canopy light distribution improved in Figure 12B since the shading problem caused by the two side walls was resolved. Comparison in Figures 13A–C against that in Figures 13D–F shows that the evenness of vertical light distribution in the three layers of the tomato canopy considerably improved. The optimal reflective film configuration contributed best to light interception in the west, then in the east, and least in the other planting areas (Table 4).

**Table 4:** Integrated compound canopy light interception gain of the final reflective film configuration scheme on December 30th.

Row numbers [West–East]	Light interception improving ratio (%)	Row numbers [West–East]	Light interception improving ratio (%)
1	31.7	21	2.6
2	16.9	22	2.1
3	10.7	23	2.1
4	6.9	24	2.2
5	4.4	25	2.3
6	3.5	26	2.2
7	2.7	27	2.1
8	2.4	28	2.1
9	2.3	29	2.3
10	2.4	30	2.3
11	2.3	31	2.3
12	2.4	32	2.4
13	2.4	33	2.7
14	2.3	34	2.8
15	2.3	35	3.4
16	2.3	36	3.6
17	2.3	37	5.1
18	2.2	38	7.7
19	2.5	39	11.7
20	2.2	40	18.9

## Discussion

The purpose of this study was to identify how supplemental reflective films affect light distribution within tomato canopy and provide a basic framework for the application of reflective films in CSG. In the case greenhouse, the 3D greenhouse model could precisely predict light radiation intensity and the horizontal and vertical light distribution has been improved under the optimal configuration compared to the scenario without reflective films.

The finds of this study are consistent with previous studies (Kong and Meng, 2004; Tang et al., 2015), where a gradually increased light enhancing effect was observed with a gradual decrease in the distance from reflective films. The findings on the place of reflective film, however, are not as exactly the same. Here, based on light distribution pattern on sunny days in low light season, reflective films were placed close to sidewalls and on the ground. In previous studies, reflective materials were applied to crops on the north side of the planting area (Kong et al., 2008), inclined north wall (Sethi and Arora, 2009) and ground (Boo et al., 2010). We surmise that this result can be explained by different study aim, wherein most studies were trying to enhance the total light interception for plants, while our study was trying to improve the light distribution within plant canopy. Another possible explanation is that there is lack of a method to precisely compute light distribution within canopy in some kind of weather condition, which could also explain the reason why the lack study of the effects of usage time and specific height of reflective films on light interception for plants.

In addition, in terms of configuration parameters of reflective films, this study not only considered the location, usage time, height, but also the inclination angle and color of the reflective film configuration. Through the simulation, it is found that tilting the reflective film on both sides of greenhouse towards sidewalls at a certain angle can effectively improve the light interception of plants (Supplementary Figure S1). However, due to the occupation of horizontal space, the reflective film inclination angle is set as zero in the final investigation of the reflective film configuration scheme. In terms of the color of reflective films (i.e., different optical properties), some studies showed that mulches with high reflectivity (i.e., white and silver) caused soil cooling at midday but kept the soil warmer than bare soil during the nighttime, due to their low transmissivity (Ham and Kluitenberg, 1994; Jones et al., 2021). Combined with low temperature at night and weak light in CSG of northern China, therefore, silver reflective film with reflectivity up to 98% was used in this study.

One of the main assumptions in the simulation process is that the individual plant traits were identical other than varied plant orientation and height above ground in the 3D greenhouse model. Although, the model validation turned out to be consistent with measured data, in reality, individual plant appears differently in terms of plant orientation, leaf area, internode length, and so on. And reflective film may slowly change the plant architecture over time by changing surrounding climate. To improve the accuracy of phenotyping traits, there are two possible solutions. One is that based on the measured database on the organ dimensions of several plants, other plants in the canopy are randomly chosen from the set of measured plants (Coussement et al., 2018). The other one is three-dimensional scanning technology for high-resolution plant phenotyping (Nguyen et al., 2016; Panjvani et al., 2019; Wang et al., 2019). And the latter one may be a promising way for timely and accurate presentation of plant canopy structure (Nguyen et al., 2016). Even though there are still unsolved problems such as expensive, not accurate for young age adult (Wang et al., 2019), difficult to form coordinates in some shaded areas (Kim et al., 2021), we believe these problems can be solved in the future.

Research on reflective films used for light distribution improvement could continue in several directions. First, microenvironment factors may be affected by supplemental reflective films, such as humidity (Mormile et al., 2017), air temperature (Wang et al., 2002), soil evaporation (Rippa et al., 2018), soil temperature (Yamaguchi et al., 1996). Then how much do the reflective films contribute to microclimate factors other than light intensity. Second, plant physiological traits, such as plant architecture, fruit setting, quality and yield, photosynthesis, and disease development and insect damage could be explored, since reflective films may change the microclimate conditions. In some studies, reflective films have been shown to improve fruit production (Meyer et al., 2012) and fruit quality (Boo et al., 2010; Overbeck et al., 2013; Jiang et al., 2014). Contrary to these studies, Lorenzo et al. (2005) found that using a highly reflective white plastic mulch, the increase in intercepted PAR by the cucumber plants did not compensate for the reduction in air/soil temperature with respect to a non-mulched soil, and that yield was lower in the mulched crop than in the non-mulched one. Therefore, a comprehensive study on the advantages and disadvantages of reflective film for specific plants would provide more information and criteria for the most appropriate reflective film configuration in CSG. Third, and perhaps most fascinating, studies could explore how to configure reflective films in other weather conditions, and could we find a compromise configuration for all kinds of weather in some stage of plants. With the latter topic, the solution would be more convenient for growers.

In conclusion, when using the framework provided here, users can compute light distribution in CSG and setup reflective films accordingly, even replace the scene in this study, which may be different geographic locations, greenhouse structure, greenhouse orientation, plant phenotype and time, etc., to provide guidance for the practical application of reflective films. Furthermore, new modular components can be integrated into the model, including crop physiological mechanisms such as photosynthesis modules, source-sink modules (Chen et al., 2014; Pallas et al., 2016), as well as new greenhouse technologies or management strategies such as greenhouse structure optimization, planting strategy optimization and plant architecture selection (Mao et al., 2016; Munz et al., 2019; Xu et al., 2020). This research and other research to follow will contribute to knowledge of the disadvantages and advantages of reflective films in CSG comprehensively, which will be good for the final decision.

## Data availability statement

The raw data supporting the conclusions of this article will be made available by the authors, without undue reservation.

## Author contributions

AL, XL, and TL planned and designed the research. AL performed experiments, conducted fieldwork, implemented the models, analyzed the data, and wrote the manuscript. MH, YZ, and DX provided methodology. MH, YL, DX, YZ, XL, and TL reviewed and edited the manuscript. XL acquired the funding. TL supervised the project. All authors contributed to the article and approved the submitted version.

## Funding

This work was supported by the National Key Research and Development Program of China (grant number 2019YFD10003001). The work of MH was supported from European Regional Development Fund-Project “SINGING PLANT” (No. CZ.02.1.01/0.0/0.0/16_026/0008446).

## Conflict of interest

The authors declare that the research was conducted in the absence of any commercial or financial relationships that could be construed as a potential conflict of interest.

## Publisher’s note

All claims expressed in this article are solely those of the authors and do not necessarily represent those of their affiliated organizations, or those of the publisher, the editors and the reviewers. Any product that may be evaluated in this article, or claim that may be made by its manufacturer, is not guaranteed or endorsed by the publisher.

## Supplementary material

SUPPLEMENTARY FIGURE S1 Daily integrated effects of the inclination angle of reflective films on tomato canopy light interception of **(A)** west side and **(B)** east side of the greenhouse on December 30th. The inclination angle of the reflective films refers to the angle of the upper end of the reflective film tilting towards sidewalls.
