# Supplementary material for: Investigation of the impact of supplemental reflective films to improve micro-light climate within tomato plant canopy in solar greenhouses
Source: Front Plant Sci. 2022 Aug 23;13:966596. doi: 10.3389/fpls.2022.966596 (PMC9445499; doi:10.3389/fpls.2022.966596)
Supplement: Supplementary file 2 [file Table_1.DOCX]

**TABLE S1**. Optical properties of different components in the case greenhouse.

|  | Absorptivity (%) | Reflectivity (%) | Transmissivity (%) | References |
| --- | --- | --- | --- | --- |
| South roof | 2.9 | 7.7 | 89.4 | (Park et al., 2021) |
| Soil | 75 | 25 | - | (Abdel-Ghany and Kozai, 2006) |
| Inside air | - | - | 100 | (Abdel-Ghany and Kozai, 2006) |
| Walls | 65 | 35 | - | (Reagan and Acklam, 1979) |
| North roof | 55 | 45 | - | (Reagan and Acklam, 1979) |
| Reflective film | - | 98 | - | (McCord et al., 2009) |
| Tomato leaf | 89 | 8 | 3 | (de Visser et al., 2014) |

Abdel-Ghany, A. M., & Kozai, T. (2006). On the determination of the overall heat transmission coefficient and soil heat flux for a fog cooled, naturally ventilated greenhouse: Analysis of radiation and convection heat transfer. *Energy Conversion and Management*, 47(15-16), 2612-2628. doi:[10.1016/j.enconman.2005.10.024](https://doi.org/10.1016/j.enconman.2005.10.024).

de Visser, P. H. B., Buck-Sorlin, G. H., and van der Heijden, G. W. A. M. (2014). Optimizing illumination in the greenhouse using a 3D model of tomato and a ray tracer. *Front. Plant Sci.* 5, 1–7. doi:10.3389/fpls.2014.00048.

McCord, K. M., Klinglesmith, D. A., Jurgenson, C. A., Bakker, E. J., Schmell, R. A., Schmell, R. A., ... & Lewis, J. (2009, August). Characterization of Silver and Aluminum custom mirror coatings for the MRO interferometric telescopes. In *Optical Materials and Structures Technologies IV* (Vol. 7425, pp. 282-290). SPIE. doi:[10.1117/12.826044](https://doi.org/10.1117/12.826044).

Park, D. Y., Lee, H. J., Yun, S. I., & Choi, S. M. (2021). Simulation Analysis of Daylight Characteristics and Cooling Load Based on Performance Test of Covering Materials Used in Smart Farms. *Energies*, 14(19), 6331. doi:[10.3390/en14196331](https://doi.org/10.3390/en14196331)

Reagan, J. A., & Acklam, D. M. (1979). Solar reflectivity of common building materials and its influence on the roof heat gain of typical southwestern USA residences. *Energy and Buildings*, 2(3), 237-248. doi:[10.1016/0378-7788(79)90009-4](https://doi.org/10.1016/0378-7788(79)90009-4).
