# Supplementary material for: Investigation of the impact of supplemental reflective films to improve micro-light climate within tomato plant canopy in solar greenhouses
Source: Front Plant Sci. 2022 Aug 23;13:966596. doi: 10.3389/fpls.2022.966596 (PMC9445499; doi:10.3389/fpls.2022.966596)
Supplement: Supplementary file 3 [file Table_2.DOCX]

**TABLE S2**. Daily solar radiation of typical sunny days in low light season of Shenyang (Meteorological Information Center, 2005). Direct solar radiation and/or global solar radiation are accumulated by corresponding hourly data during 9:00-16:00 on December 30th in typical meteorological year.

| Date | Direct solar radiation (W m^-2^) | Global solar radiation (W m^-2^) | Direct solar radiation/Global solar radiation (%) |
| --- | --- | --- | --- |
| November 6th | 1883 | 2714 | 69 |
| December 30th | 1533 | 2084 | 74 |
| January 29th | 1903 | 2533 | 75 |
| February 26th | 2759 | 3953 | 70 |
